# Crystal structure of (1*R*,3*S*,8*R*,11*R*)-11-acetyl-3,7,7-trimethyl-10-oxatri­cyclo­[6.4.0.0^1,3^]dodecan-9-one

**DOI:** 10.1107/S2056989015022847

**Published:** 2015-12-06

**Authors:** Abdoullah Bismoussa, My Youssef Ait Itto, Jean-Claude Daran, Abdelwahed Auhmani, Aziz Auhmani

**Affiliations:** a Laboratoire de Physico-Chimie Moléculaire et Synthèse Organique, Département de Chimie Faculté des Sciences Semlalia BP, 2390 Marrakech 40001, Morocco; b Laboratoire de Chimie de Coordination, 205 route de Narbonne, 31077 Toulouse, Cedex 04, France

**Keywords:** Lactones, fused rings, biological activities, crystal structure

## Abstract

The title compound, C_16_H_24_O_3_, is built up from three fused rings, a six-membered, a seven-membered and a three-membered ring. The absolute configuration of the title compound was determined as (1*R*,3S,8*R*,11*R*) based on the synthetic pathway. The six-membered ring has an half-chair conformation whereas the seven-membered ring displays a boat conformation. In the cyrstal, C—H⋯O hydrogen bonds build up a two-dimensional network parallel to (0 0 1). The crystal studied was an inversion twin with a minor twin component of 34%.

## Related literature   

For biological activities of terpenic lactones, see: Hall *et al.* (1987 ▸); Ohnishi *et al.* (1997 ▸); Ghosh & Karin (2002 ▸); Bremner & Heinrich (2002 ▸); Francois *et al.* (1996 ▸); Rabe *et al.* (2002 ▸); Calera *et al.* (1995 ▸). For the synthesis, see: Bimoussa *et al.* (2014 ▸). For the ring puckering parameters, see: Boessenkool & Boyens (1980 ▸). For the absolute configuration, see: Parsons *et al.* (2013 ▸); Hooft *et al.* (2008 ▸). For the refinement of twined crystals, see: Cooper *et al.* (2002 ▸).
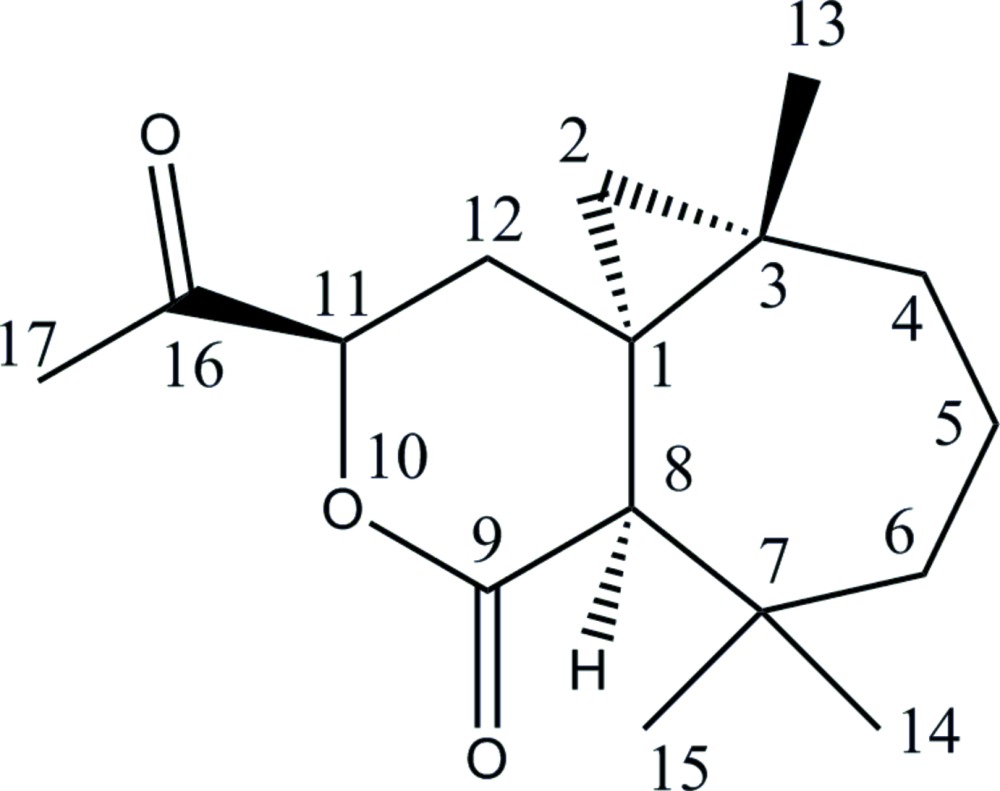


## Experimental   

### Crystal data  

C_16_H_24_O_3_
*M* *_r_* = 264.35
Monoclinic, 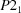

*a* = 6.4443 (6) Å
*b* = 8.4437 (7) Å
*c* = 13.7083 (12) Å
β = 98.654 (9)°
*V* = 737.43 (11) Å^3^
*Z* = 2
Mo *K*α radiation
μ = 0.08 mm^−1^
*T* = 180 K
0.52 × 0.45 × 0.25 mm

### Data collection  

Agilent Xcalibur (Eos, Gemini ultra) diffractometer
Absorption correction: multi-scan (*CrysAlis PRO*; Agilent, 2014 ▸). *T*_min_ = 0.717, *T*_max_ = 1.000
7878 measured reflections
7878 independent reflections
7173 reflections with *I* > 2σ(*I*)
*R*_int_ = 0.041

### Refinement  

*R*[*F* ^2^ > 2σ(*F* ^2^)] = 0.061
*wR*(*F* ^2^) = 0.141
*S* = 1.05
7878 reflections
177 parameters
1 restraint
H-atom parameters constrained
Δρ_max_ = 0.31 e Å^−3^
Δρ_min_ = −0.34 e Å^−3^

### 

Data collection: *CrysAlis PRO* (Agilent, 2014 ▸); cell refinement: *CrysAlis PRO*; data reduction: *CrysAlis PRO*; program(s) used to solve structure: *SIR97* (Altomare *et al.*, 1999 ▸); program(s) used to refine structure: *SHELXL2013* (Sheldrick, 2015 ▸); molecular graphics: *ORTEPIII* (Burnett & Johnson, 1996 ▸) and *ORTEP-3 for Windows* (Farrugia, 2012 ▸); software used to prepare material for publication: *SHELXL2013*.

## Supplementary Material

Crystal structure: contains datablock(s) I, New_Global_Publ_Block. DOI: 10.1107/S2056989015022847/xu5880sup1.cif

Structure factors: contains datablock(s) I. DOI: 10.1107/S2056989015022847/xu5880Isup2.hkl

Supporting information file. DOI: 10.1107/S2056989015022847/xu5880Isup3.cml

**Fig. 1.**
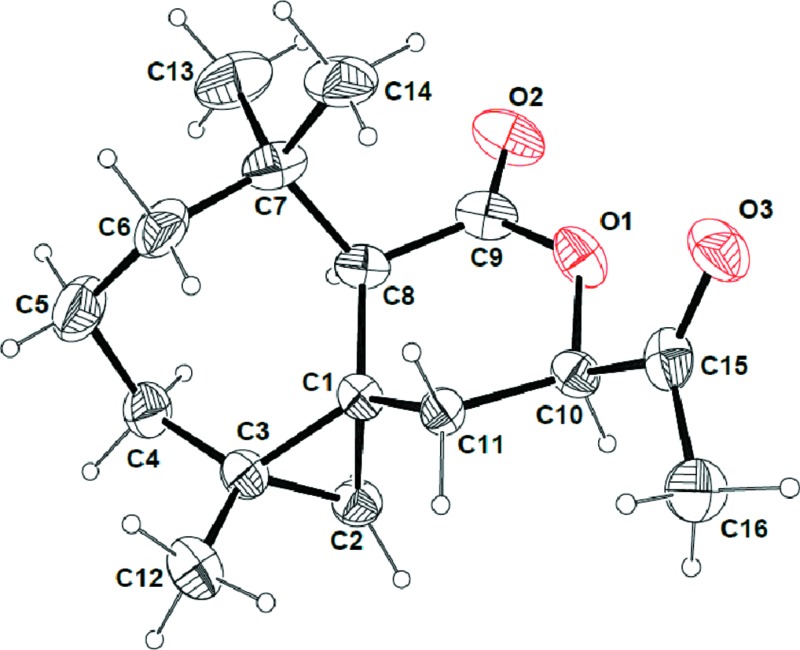
Molecular view of the title compound with the atom-labeling scheme. Displacement ellipsoids are drawn at the 50% probability level. H atoms are represented as small circle of arbitrary radii.

. DOI: 10.1107/S2056989015022847/xu5880fig1.tif
Mol­ecular view of the title compound with the atom-labeling scheme. Displacement ellipsoids are drawn at the 50% probability level. H atoms are represented as small circle of arbitrary radii.

**Fig. 2.**
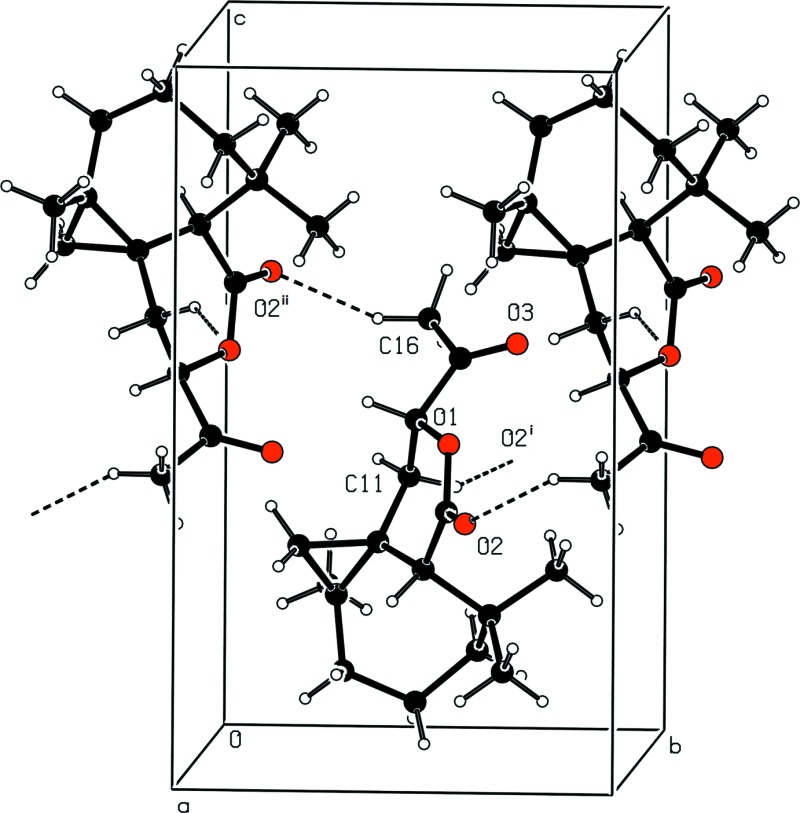
Packing view showing the C—H···O hydrogen bonds building a two-dimensional network. H bonds are shown as dashed lines. [Symmetry codes: (i) x + 1, y, z; (ii) -x + 1, y + 1/2, -z + 1].

Click here for additional data file.x y z x y z . DOI: 10.1107/S2056989015022847/xu5880fig2.tif
Packing view showing the C—H⋯O hydrogen bonds building a two-dimensional network. H bonds are shown as dashed lines. [Symmetry codes: (i) *x* + 1, *y*, *z*; (ii) −*x* + 1, *y* + 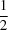, −*z* + 1].

CCDC reference: 1439412

Additional supporting information:  crystallographic information; 3D view; checkCIF report

## Figures and Tables

**Table 1:** Hydrogen-bond geometry (Å, °)

*D*—H⋯*A*	*D*—H	H⋯*A*	*D*⋯*A*	*D*—H⋯*A*
C11—H11*A*⋯O2^i^	0.99	2.40	3.184 (6)	136
C16—H16*C*⋯O2^ii^	0.98	2.37	3.262 (8)	152

Symmetry codes: (i) 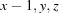; (ii) 
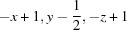
.

## supplementary crystallographic information

### S1. Chemical context

In recent years many terpenic lactones were shown to exhibit broad spectrum of biological activities such anti­cancer activity (Hall *et al.*, 1987, Ohnishi *et al.*, 1997), anti-inflammatory activity (Ghosh & Karin, 2002; Bremner & Heinrich, 2002), anti-malarial activity (Francois *et al.*, 1996), anti­viral activity, anti­bacterial activity (Rabe *et al.*, 2002) and anti­fungal activity (Calera *et al.*, 1995).

Their structural diversity and potential biological activities have made further inter­est for the drug discovery research. Thus, In order to prepare new lactones using natural products, we have prepared (1R,3S,8R,11R)-11-acetyl-3,7,7-tri­methyl-10-oxatri­cyclo­[6.4.0.0^1,3^]dodecan-9-one from β-himachalène (sesquiterpenic hydro­carbon). The title compound was prepared by an oxidative cleavage of (1S,3S,8R,9S,10R)-9,10-Ep­oxy-3,7,7,10-tetra­methyl­tri­cyclo­[6.4.0.0^1,3^]do­decane (Bimoussa *et al.*, 2014) using periodic acid as oxydant.

### S2. Structural commentary

The compound is built up from three fused rings, a six membered and a seven membered rings; these last ring is fused with a three membered ring (Fig. 1). The six-membered-ring has an half chair conformation with puckering parameters: Q = 0.469 (3) Å, θ = 39.3 (4)° and φ = 213.6 (6)°, whereas the seven-membered ring displays a boat conformation with puckering amplitudes: Q2 = 1.130 (4) and Q3 =0.044 (4) (Boessenkool & Boyens, 1980).

The absolute configuration (1S,3R,8S,10S) is deduced from the chemical pathway. The refinement of the Flack's parameter (-0.0 (10)) (Parsons *et al.*, 2013) as well as the Hooft's parameter (Hooft *et al.*, 2008) do not allow to define reliably the absolute configuration.

### S3. Supra­molecular features

There are weak C—H···O hydrogen bonds building a two dimensional network parallel to the (0 0 1) plane (Fig. 2).

### S4. Database survey

A search of the Cambridge Structural Database gave no hits with related structures.

### S5. Synthesis and crystallization

In 100 ml flask containing (0.120g, (0.515 mmol) of (1*S*,3*S*,8*R*,9*S*,10*R*)-9,10-Ep­oxy-3,7,7,10-tetra­methyl­tri­cyclo­[6.4.0.0^1,3^]do­decane in 6ml of CCl_4_, 6 ml of aceto­nitrile and 6ml of watter was added 0.427 g (2.06 mmol) of periodic acid (*NaIO_4_*) and 4.70 mg (0.0179 mmol) of RuCl_3_,3H_2_O ( 3.5%). The reaction mixture was stirred at 0°C for 20 min and for 24h at room temperature. The reaction mixture was extracted with di­chloro­methane (3x 20ml) and the organic layer were dried over Na_2_SO_4_ and concentrated under reduced pressure. The crude product was purified by chromatography on silica gel (230–400 mesh) with Hexane/ethyl acetate (85:15) as eluent to give the title compound (1R,3S,8R,11R)-11-acetyl-3,7,7-tri­methyl-10-oxatri­cyclo­[6.4.0.0^1,3^]dodecan-9-one in 46% yield. X-ray quality crystals were obtained by slow evaporation from a petroleum ether solution of the title compound.

### S6. Refinement details

The crystal is twinned and has been refined as a 2-component twin with the following matrix using ROTAX (Parsons 1 Gould; Cooper *et al.*, 2002): 180.0 degree rotation about 1. 0. 0. direct lattice direction: [ 1.000 0.000 0.000] [ 0.000 -1.000 0.000] [ -0.640 0.000 -1.000] BASF = 0.34

### Crystal data

**Table:**

C_16_H_24_O_3_	*F*(000) = 288
*M**_r_* = 264.35	*D*_x_ = 1.191 Mg m^−^^3^
Monoclinic, *P*2_1_	Mo *K*α radiation, λ = 0.71073 Å
*a* = 6.4443 (6) Å	Cell parameters from 2697 reflections
*b* = 8.4437 (7) Å	θ = 4.0–28.4°
*c* = 13.7083 (12) Å	µ = 0.08 mm^−^^1^
β = 98.654 (9)°	*T* = 180 K
*V* = 737.43 (11) Å^3^	Box, colourless
*Z* = 2	0.52 × 0.45 × 0.25 mm

### Data collection

**Table:**

Agilent Xcalibur (Eos, Gemini ultra) diffractometer	7878 independent reflections
Radiation source: Enhance (Mo) X-ray Source	7173 reflections with *I* > 2σ(*I*)
Graphite monochromator	*R*_int_ = 0.041
Detector resolution: 16.1978 pixels mm^-1^	θ_max_ = 26.4°, θ_min_ = 3.0°
ω scans	*h* = −8→8
Absorption correction: multi-scan (*CrysAlis PRO*; Agilent, 2014).	*k* = −10→10
*T*_min_ = 0.717, *T*_max_ = 1.000	*l* = −17→16
7878 measured reflections	

### Refinement

**Table:**

Refinement on *F*^2^	1 restraint
Least-squares matrix: full	Hydrogen site location: inferred from neighbouring sites
*R*[*F*^2^ > 2σ(*F*^2^)] = 0.061	H-atom parameters constrained
*wR*(*F*^2^) = 0.141	*w* = 1/[σ^2^(*F*_o_^2^) + (0.0238*P*)^2^ + 0.9242*P*] where *P* = (*F*_o_^2^ + 2*F*_c_^2^)/3
*S* = 1.05	(Δ/σ)_max_ < 0.001
7878 reflections	Δρ_max_ = 0.31 e Å^−^^3^
177 parameters	Δρ_min_ = −0.34 e Å^−^^3^

### Special details

**Table:**

Geometry. All e.s.d.'s (except the e.s.d. in the dihedral angle between two l.s. planes) are estimated using the full covariance matrix. The cell e.s.d.'s are taken into account individually in the estimation of e.s.d.'s in distances, angles and torsion angles; correlations between e.s.d.'s in cell parameters are only used when they are defined by crystal symmetry. An approximate (isotropic) treatment of cell e.s.d.'s is used for estimating e.s.d.'s involving l.s. planes.

### Fractional atomic coordinates and isotropic or equivalent isotropic displacement parameters (Å^2^)

**Table:**

	*x*	*y*	*z*	*U*_iso_*/*U*_eq_	
C1	0.2887 (8)	0.3804 (6)	0.2813 (4)	0.0237 (11)	
C2	0.3418 (9)	0.2072 (6)	0.2798 (4)	0.0297 (12)	
H2A	0.2894	0.1378	0.3289	0.036*	
H2B	0.4811	0.1776	0.2634	0.036*	
C3	0.1834 (8)	0.2745 (7)	0.1985 (4)	0.0274 (12)	
C4	0.2531 (10)	0.2989 (7)	0.0992 (4)	0.0359 (14)	
H4A	0.2061	0.2079	0.0559	0.043*	
H4B	0.4084	0.3023	0.1078	0.043*	
C5	0.1648 (11)	0.4526 (8)	0.0492 (4)	0.0482 (17)	
H5A	0.0218	0.4315	0.0140	0.058*	
H5B	0.2534	0.4840	−0.0006	0.058*	
C6	0.1541 (10)	0.5920 (7)	0.1208 (4)	0.0402 (15)	
H6A	0.1000	0.6852	0.0811	0.048*	
H6B	0.0487	0.5650	0.1637	0.048*	
C7	0.3540 (9)	0.6425 (7)	0.1873 (4)	0.0378 (14)	
C8	0.4454 (8)	0.5023 (6)	0.2584 (4)	0.0278 (13)	
H8	0.5466	0.4443	0.2228	0.033*	
C9	0.5737 (8)	0.5692 (7)	0.3498 (4)	0.0319 (13)	
C10	0.3256 (8)	0.4700 (7)	0.4541 (4)	0.0266 (12)	
H10	0.3797	0.3666	0.4830	0.032*	
C11	0.1686 (8)	0.4366 (6)	0.3621 (3)	0.0234 (11)	
H11A	0.0888	0.5340	0.3408	0.028*	
H11B	0.0680	0.3541	0.3762	0.028*	
C12	−0.0452 (8)	0.2292 (7)	0.1912 (4)	0.0371 (15)	
H12A	−0.0664	0.1231	0.1623	0.056*	
H12B	−0.1315	0.3057	0.1493	0.056*	
H12C	−0.0860	0.2292	0.2572	0.056*	
C13	0.5222 (11)	0.6924 (9)	0.1249 (5)	0.061 (2)	
H13A	0.6470	0.7301	0.1684	0.091*	
H13B	0.4669	0.7775	0.0797	0.091*	
H13C	0.5597	0.6014	0.0868	0.091*	
C14	0.2968 (11)	0.7879 (7)	0.2460 (5)	0.0483 (17)	
H14A	0.2497	0.8743	0.2002	0.072*	
H14B	0.4204	0.8221	0.2917	0.072*	
H14C	0.1839	0.7596	0.2834	0.072*	
C15	0.2174 (8)	0.5569 (7)	0.5301 (4)	0.0301 (12)	
C16	0.0480 (9)	0.4660 (7)	0.5691 (4)	0.0372 (14)	
H16A	−0.0855	0.4816	0.5254	0.056*	
H16B	0.0349	0.5037	0.6355	0.056*	
H16C	0.0836	0.3531	0.5718	0.056*	
O1	0.5017 (6)	0.5651 (5)	0.4367 (3)	0.0370 (10)	
O2	0.7419 (6)	0.6310 (6)	0.3489 (3)	0.0476 (11)	
O3	0.2640 (7)	0.6912 (5)	0.5555 (3)	0.0415 (10)	

### Atomic displacement parameters (Å^2^)

**Table:**

	*U*^11^	*U*^22^	*U*^33^	*U*^12^	*U*^13^	*U*^23^
C1	0.021 (3)	0.023 (3)	0.027 (3)	0.002 (2)	0.003 (2)	−0.003 (2)
C2	0.034 (3)	0.023 (3)	0.033 (3)	0.003 (2)	0.006 (3)	−0.001 (3)
C3	0.029 (3)	0.025 (3)	0.029 (3)	0.002 (2)	0.005 (2)	−0.005 (2)
C4	0.044 (3)	0.037 (4)	0.027 (3)	0.000 (3)	0.007 (3)	−0.005 (3)
C5	0.069 (5)	0.048 (4)	0.028 (3)	0.006 (4)	0.009 (3)	0.005 (3)
C6	0.053 (4)	0.032 (3)	0.034 (3)	0.012 (3)	0.002 (3)	0.011 (3)
C7	0.043 (3)	0.027 (3)	0.047 (3)	0.004 (3)	0.019 (3)	0.006 (3)
C8	0.026 (3)	0.024 (3)	0.037 (3)	0.000 (2)	0.014 (3)	−0.003 (2)
C9	0.018 (2)	0.027 (3)	0.052 (3)	0.004 (2)	0.010 (3)	−0.002 (3)
C10	0.026 (3)	0.023 (3)	0.030 (3)	0.001 (2)	0.003 (2)	−0.003 (2)
C11	0.022 (2)	0.027 (3)	0.022 (2)	−0.002 (2)	0.007 (2)	−0.002 (2)
C12	0.031 (3)	0.039 (4)	0.040 (3)	0.001 (3)	0.002 (3)	−0.009 (3)
C13	0.067 (5)	0.056 (5)	0.067 (4)	−0.008 (4)	0.032 (4)	0.019 (4)
C14	0.059 (4)	0.021 (3)	0.064 (4)	0.006 (3)	0.008 (4)	0.004 (3)
C15	0.031 (3)	0.030 (3)	0.027 (3)	0.013 (3)	−0.003 (2)	−0.001 (3)
C16	0.041 (3)	0.036 (3)	0.038 (3)	0.001 (3)	0.018 (3)	−0.006 (3)
O1	0.0256 (19)	0.043 (2)	0.041 (2)	−0.004 (2)	0.0022 (18)	−0.013 (2)
O2	0.023 (2)	0.044 (3)	0.077 (3)	−0.008 (2)	0.010 (2)	−0.011 (2)
O3	0.050 (3)	0.030 (2)	0.043 (2)	0.001 (2)	0.003 (2)	−0.012 (2)

### Geometric parameters (Å, º)

**Table:**

C1—C2	1.503 (7)	C9—O2	1.205 (6)
C1—C8	1.507 (7)	C9—O1	1.343 (6)
C1—C11	1.520 (7)	C10—O1	1.438 (6)
C1—C3	1.523 (7)	C10—C11	1.519 (7)
C2—C3	1.505 (7)	C10—C15	1.527 (7)
C2—H2A	0.9900	C10—H10	1.0000
C2—H2B	0.9900	C11—H11A	0.9900
C3—C12	1.511 (7)	C11—H11B	0.9900
C3—C4	1.511 (7)	C12—H12A	0.9800
C4—C5	1.537 (9)	C12—H12B	0.9800
C4—H4A	0.9900	C12—H12C	0.9800
C4—H4B	0.9900	C13—H13A	0.9800
C5—C6	1.541 (8)	C13—H13B	0.9800
C5—H5A	0.9900	C13—H13C	0.9800
C5—H5B	0.9900	C14—H14A	0.9800
C6—C7	1.523 (8)	C14—H14B	0.9800
C6—H6A	0.9900	C14—H14C	0.9800
C6—H6B	0.9900	C15—O3	1.211 (7)
C7—C13	1.537 (8)	C15—C16	1.498 (8)
C7—C14	1.542 (8)	C16—H16A	0.9800
C7—C8	1.590 (7)	C16—H16B	0.9800
C8—C9	1.503 (7)	C16—H16C	0.9800
C8—H8	1.0000		
			
C2—C1—C8	120.0 (5)	C1—C8—H8	105.9
C2—C1—C11	117.1 (5)	C7—C8—H8	105.9
C8—C1—C11	111.7 (4)	O2—C9—O1	116.9 (5)
C2—C1—C3	59.6 (3)	O2—C9—C8	122.5 (5)
C8—C1—C3	118.9 (5)	O1—C9—C8	120.6 (4)
C11—C1—C3	120.5 (4)	O1—C10—C11	114.2 (4)
C1—C2—C3	60.8 (4)	O1—C10—C15	107.3 (4)
C1—C2—H2A	117.7	C11—C10—C15	109.9 (4)
C3—C2—H2A	117.7	O1—C10—H10	108.4
C1—C2—H2B	117.7	C11—C10—H10	108.4
C3—C2—H2B	117.7	C15—C10—H10	108.4
H2A—C2—H2B	114.8	C10—C11—C1	108.3 (4)
C2—C3—C12	120.0 (5)	C10—C11—H11A	110.0
C2—C3—C4	117.3 (5)	C1—C11—H11A	110.0
C12—C3—C4	113.2 (5)	C10—C11—H11B	110.0
C2—C3—C1	59.5 (3)	C1—C11—H11B	110.0
C12—C3—C1	121.3 (4)	H11A—C11—H11B	108.4
C4—C3—C1	115.6 (5)	C3—C12—H12A	109.5
C3—C4—C5	112.1 (5)	C3—C12—H12B	109.5
C3—C4—H4A	109.2	H12A—C12—H12B	109.5
C5—C4—H4A	109.2	C3—C12—H12C	109.5
C3—C4—H4B	109.2	H12A—C12—H12C	109.5
C5—C4—H4B	109.2	H12B—C12—H12C	109.5
H4A—C4—H4B	107.9	C7—C13—H13A	109.5
C4—C5—C6	114.2 (4)	C7—C13—H13B	109.5
C4—C5—H5A	108.7	H13A—C13—H13B	109.5
C6—C5—H5A	108.7	C7—C13—H13C	109.5
C4—C5—H5B	108.7	H13A—C13—H13C	109.5
C6—C5—H5B	108.7	H13B—C13—H13C	109.5
H5A—C5—H5B	107.6	C7—C14—H14A	109.5
C7—C6—C5	118.7 (5)	C7—C14—H14B	109.5
C7—C6—H6A	107.6	H14A—C14—H14B	109.5
C5—C6—H6A	107.6	C7—C14—H14C	109.5
C7—C6—H6B	107.6	H14A—C14—H14C	109.5
C5—C6—H6B	107.6	H14B—C14—H14C	109.5
H6A—C6—H6B	107.1	O3—C15—C16	122.7 (5)
C6—C7—C13	110.3 (5)	O3—C15—C10	121.8 (5)
C6—C7—C14	106.7 (5)	C16—C15—C10	115.4 (5)
C13—C7—C14	108.4 (5)	C15—C16—H16A	109.5
C6—C7—C8	111.1 (5)	C15—C16—H16B	109.5
C13—C7—C8	108.5 (5)	H16A—C16—H16B	109.5
C14—C7—C8	111.6 (5)	C15—C16—H16C	109.5
C9—C8—C1	112.6 (4)	H16A—C16—H16C	109.5
C9—C8—C7	109.7 (5)	H16B—C16—H16C	109.5
C1—C8—C7	116.0 (4)	C9—O1—C10	123.2 (4)
C9—C8—H8	105.9		

### Hydrogen-bond geometry (Å, º)

**Table:**

*D*—H···*A*	*D*—H	H···*A*	*D*···*A*	*D*—H···*A*
C11—H11*A*···O2^i^	0.99	2.40	3.184 (6)	136
C16—H16*C*···O2^ii^	0.98	2.37	3.262 (8)	152

Symmetry codes: (i) *x*−1, *y*, *z*; (ii) −*x*+1, *y*−1/2, −*z*+1.
