# Supplementary figures and images for: Crystal structure of (1R,3S,8R,11R)-11-acetyl-3,7,7-trimethyl-10-oxatri­cyclo­[6.4.0.01,3]dodecan-9-one
Source: Acta Crystallogr E Crystallogr Commun. 2015 Dec 6;71(Pt 12):o1013–4. doi: 10.1107/S2056989015022847 (PMC4719952; doi:10.1107/S2056989015022847)

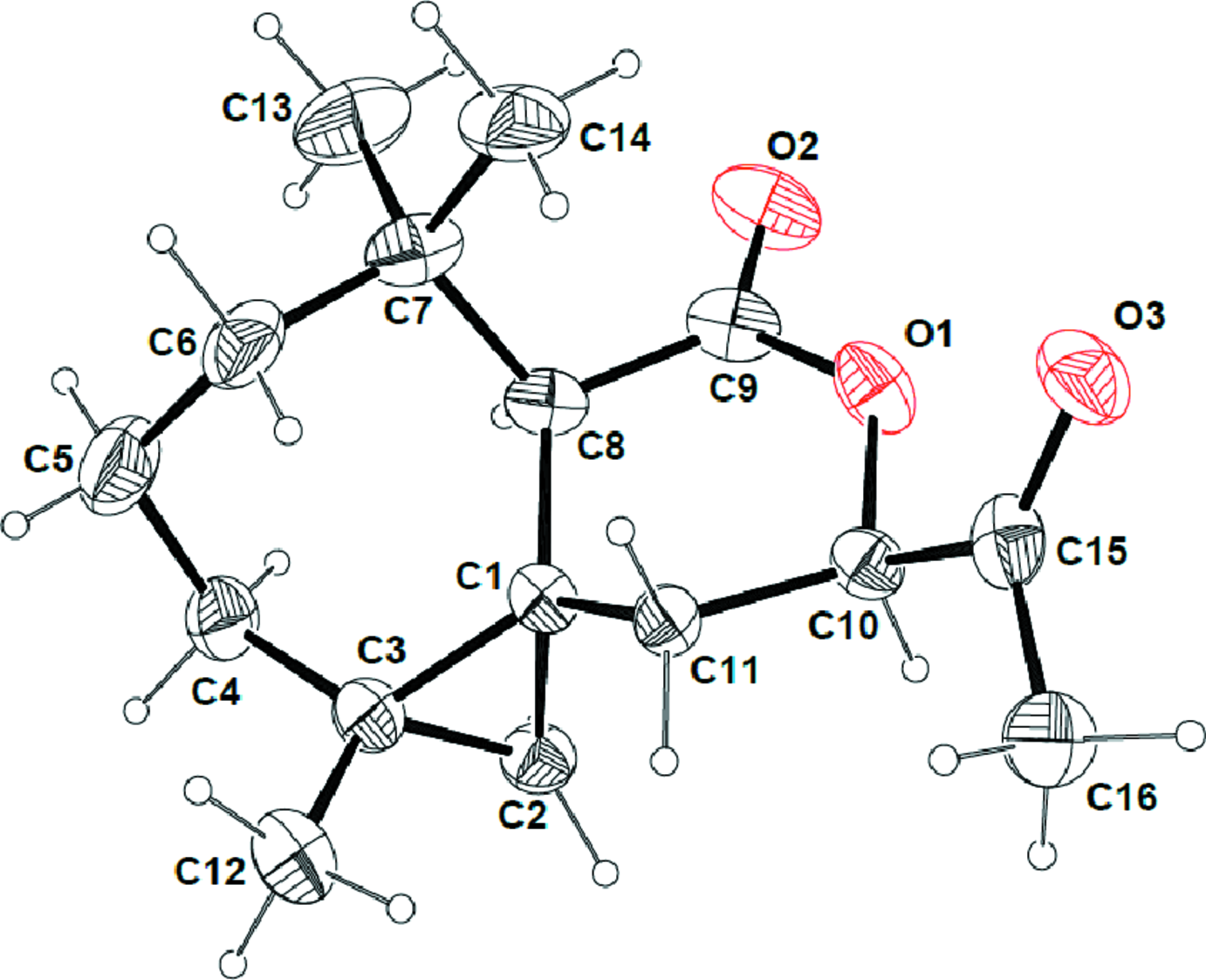

Supplement: Supplementary file 4 [file e-71-o1013-fig1.tif]

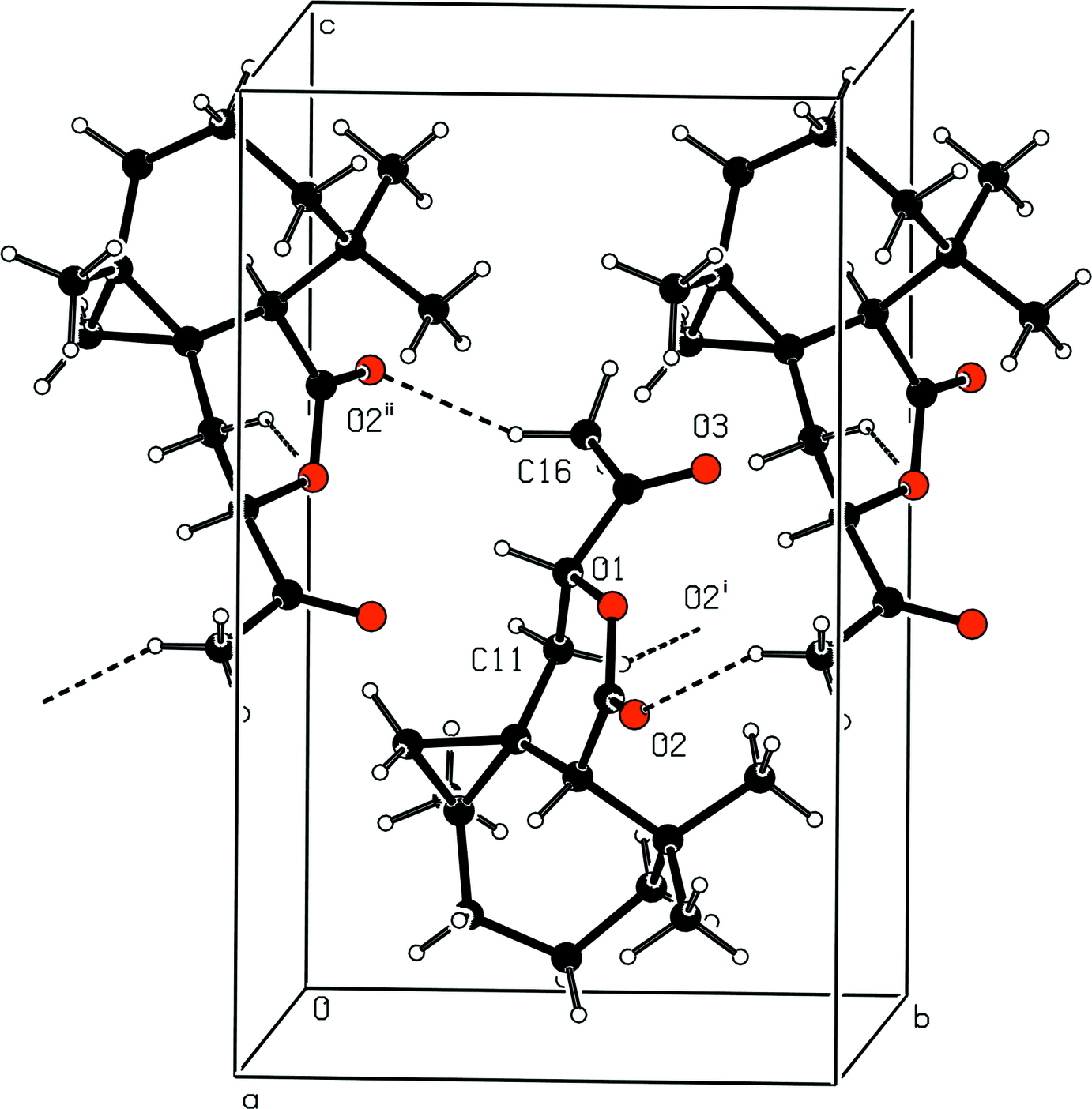

Supplement: Supplementary file 5 [file e-71-o1013-fig2.tif]
